# Supplementary material for: EnzML: multi-label prediction of enzyme classes using InterPro signatures
Source: BMC Bioinformatics. 2012 Apr 25;13:61. doi: 10.1186/1471-2105-13-61 (PMC3483700; doi:10.1186/1471-2105-13-61)
Supplement: Addtional file 5 — The Java code to format the data files, evaluate and predict. The file enzml_java_code.tar.gz contains the Java code used to format database data to ARFF and XML formats, to execute cross and train-test (jackknife) evaluations and to record evaluation results to database. More information is included in the readme.txt file and the Javadoc files. The code can be used with a MySQL database. To use a different database software, other JDBC drivers might be required. [file 1471-2105-13-61-S5.gz › java_code/enzml2011/doc/uk/ac/ed/inf/enzml/mulan/MulanDataSetManager.html]

MulanDataSetManager


---


|  |  |  |
| --- | --- | --- |
| **Overview** \| **Package** \| **Class** \| **Use** \| **Tree** \| **Deprecated** \| **Index** \| **Help** | |  |
| **PREV CLASS**   **NEXT CLASS** | **FRAMES**    **NO FRAMES**     **All Classes** |  |
| SUMMARY: NESTED \| FIELD \| CONSTR \| METHOD | DETAIL: FIELD \| CONSTR \| METHOD |  |


---


## uk.ac.ed.inf.enzml.mulan Class MulanDataSetManager

```
java.lang.Object
  uk.ac.ed.inf.utils.database.Manager
      uk.ac.ed.inf.enzml.weka.DataSetManager
          uk.ac.ed.inf.enzml.mulan.MulanDataSetManager
```

**Direct Known Subclasses:**: AttributesFilteredDataSetManager

---

``` public class MulanDataSetManager extends DataSetManager ```

This class generates a data set (instances, attributes, classes) in Mulan
style (for multi-label machine learning). The main difference compared with
the classic Weka ARFF, is that there are multiple classes and each is listed
as a normal binary {0, 1} attribute. In order to distinguish the normal
attributes from the class attributes, the class attributes values must also
be specified in a separate Mulan XML.

**Version:**
:   7 May 2010

**Author:**
:   Luna De Ferrari luna.deferrari-at-ed.ac.uk

---

**Field Summary**

**Fields inherited from class uk.ac.ed.inf.enzml.weka.DataSetManager**

`DUMMY_VALUE`


**Constructor Summary**

`MulanDataSetManager(MulanArff arff)`


| **Method Summary** | |
| --- | --- |
| `MulanArff` | `getArffGenerator()` |
| `MulanDataSetDbLoader` | `getDataLoader()` |
| `MulanDataSetGenerator` | `getDataSetGenerator()` |

**Methods inherited from class uk.ac.ed.inf.enzml.weka.DataSetManager**

`fillArffButDontWrite, getArffProperties, getClassName, getDataSetChecker, getDataSetName, getDataSetWriter, initialisationIsCorrect, writeArff`

**Methods inherited from class java.lang.Object**

`equals, getClass, hashCode, notify, notifyAll, toString, wait, wait, wait`

**Constructor Detail**

### MulanDataSetManager

```
public MulanDataSetManager(MulanArff arff)
```


**Method Detail**

### getArffGenerator

```
public MulanArff getArffGenerator()
```

:   **Overrides:**: `getArffGenerator` in class `DataSetManager`

---


### getDataLoader

```
public MulanDataSetDbLoader getDataLoader()
```

:   **Overrides:**: `getDataLoader` in class `DataSetManager`

---


### getDataSetGenerator

```
public MulanDataSetGenerator getDataSetGenerator()
```

:   **Overrides:**: `getDataSetGenerator` in class `DataSetManager`


---


|  |  |  |
| --- | --- | --- |
| **Overview** \| **Package** \| **Class** \| **Use** \| **Tree** \| **Deprecated** \| **Index** \| **Help** | |  |
| **PREV CLASS**   **NEXT CLASS** | **FRAMES**    **NO FRAMES**     **All Classes** |  |
| SUMMARY: NESTED \| FIELD \| CONSTR \| METHOD | DETAIL: FIELD \| CONSTR \| METHOD |  |


---
